# Halogenated tryptophan derivatives disrupt essential transamination mechanisms in bloodstream form *Trypanosoma brucei*

**DOI:** 10.1371/journal.pntd.0008928

**Published:** 2020-12-04

**Authors:** Peter E. Cockram, Emily A. Dickie, Michael P. Barrett, Terry K. Smith

**Affiliations:** 1 Biomedical Sciences Research Complex, University of St Andrews, North Haugh, St Andrews, Scotland; 2 Wellcome Centre for Integrative Parasitology, Institute of Infection, Immunity and Inflammation, University of Glasgow, Glasgow, United Kingdom; University of Utah, UNITED STATES

## Abstract

Amino acid metabolism within *Trypanosoma brucei*, the causative agent of human African trypanosomiasis, is critical for parasite survival and virulence. Of these metabolic processes, the transamination of aromatic amino acids is one of the most important. In this study, a series of halogenated tryptophan analogues were investigated for their anti-parasitic potency. Several of these analogues showed significant trypanocidal activity. Metabolomics analysis of compound-treated parasites revealed key differences occurring within aromatic amino acid metabolism, particularly within the widely reported and essential transamination processes of this parasite.

## Author summary

The uptake and metabolism of amino acids, particularly the aromatic amino acids tryptophan, phenylalanine and tyrosine, is greatly important to the survival of *Trypanosoma brucei*, the causative agent of human African trypanosomiasis (HAT). Substantial differences in amino acid metabolism between parasite and human host cells provide a potential route to selective chemotherapeutic agents, which has previously been exploited in the case of the frontline HAT drug eflornithine. We have investigated analogues of the amino acid tryptophan and found that several analogues displayed selective trypanocidal potency, which strongly correlated to a structural modification of halogenation at the 7-position of the indole ring. The trypanocidal activity of these potent compounds could be reversed by supplementing growth media with excess levels of natural tryptophan, suggesting competition and direct involvement of the compounds in tryptophan metabolism. Investigation of parasite metabolism when treated with potent analogues revealed large disturbances of the parasites’ metabolic function. Importantly, significant disruptions to the metabolism of aromatic amino acids were observed, giving evidence for the direct disruption of this pathway by these compounds. In the future, these analogues could serve as vital tools to gain a deeper understanding of the functional significance of these transamination processes within *T*. *brucei* parasites.

## Introduction

The trypanosomatids are a group of biochemically related protozoan parasites, which cause a wide range of both human and animal disease across the world [1]. African trypanosomatids are endemic in sub-Saharan Africa, where they pose a significant risk to human life and inflict economic losses of up to $4.5 billion per year through animal infection [2–6]. The two human-infective sub-species of *Trypanosoma brucei* are transmitted by the tsetse fly (*Glossina* sp.) and cause different forms of Human African Trypanosomiasis (HAT). *T*. *b*. *gambiense* causes a slow-onset, chronic form of the disease throughout Western and Central Africa; while *T*. *b*. *rhodesiense* causes a rapid, acute form of the disease in Eastern and Southern Africa. Current treatments for HAT are inadequate and severely outdated, with many showing unacceptable side-effects and mounting parasite resistance [7,8]. The World Health Organization (WHO) has reported a drastic need for the development of new affordable and efficacious drugs for the treatment of these diseases [9]. Whilst significant progress has been made in recent years, a sustained effort is required to maintain disease control and the efficacy of new drugs now entering the field if elimination targets are to be met.

Amino acid uptake is of paramount importance to trypanosomatid parasites and *T*. *brucei* is auxotrophic for a number of amino acids [10–12]. In addition to their functional use in protein synthesis, *T*. *brucei* engages in the complex metabolism of amino acids for different cellular functions across the different morphologies of its life cycle. A recently published review describes, in significant detail, the uptake and metabolism of amino acids in trypanosomatids. [13] The frontline drug eflornithine is a non-natural amino acid analogue that targets amino acid metabolism in *T*. *brucei* for the treatment of HAT. Eflornithine prevents the metabolism of ornithine within *T*. *brucei* by covalently inhibiting ornithine decarboxylase [14,15]. While Eflornithine may successfully inactivate both trypanosomal and human enzymes, the drug achieves good species selectivity through differences in enzyme turnover rate [16]. Amino acid metabolism is, therefore, a validated target for the treatment of trypanosomatid-derived disease, with potential for high parasite specificity.

The rapid consumption and turnover of the aromatic amino acids tryptophan, phenylalanine and tyrosine by *T*. *brucei* is one of the most important and widely studied aspects of amino acid metabolism by trypanosomatids [17–19]. It is well reported that these aromatic amino acids are depleted from host plasma during trypanosome infection [20–22] and *T*. *brucei* converts exogeneous sources of these amino acids to their corresponding α-ketoacids indolepyruvate, phenylpyruvate and 4-hydroxyphenylpyruvate that are mostly excreted by the parasite and, in turn, by the host [20,23–25]. While the functional significance of this transamination process is not fully understood, it has been suggested that the excreted products are responsible for some of the second stage neurological symptoms of HAT [21,22] and/or help potentiate *T*. *brucei* infections by reducing the effectiveness of the host’s innate immune system [26].

Given the importance of the uptake and metabolism of aromatic amino acids in *T*. *brucei* and the established use of eflornithine in the treatment of HAT, we sought to investigate non-natural tryptophan analogues for their trypanocidal activity and mode of action.

## Methods

### Cell culture

Bloodstream trypomastigote form *Trypanosoma brucei brucei* Lister 427 strain were grown at 37°C in HMI-11 medium [27] supplemented with 10% heat-inactivated foetal bovine serum (HI-FBS, Sigma) and 2.5 μg/mL G418 (Calbiochem) with 5% v/v CO_2_ atmosphere. Cells were passaged every 2–3 days to maintain mid-log phase.

Procyclic trypomastigote form *Trypanosoma brucei brucei* strain 29–13 were grown at 28°C in SDM-79 medium [28] (Gibco) supplemented with 10% heat-inactivated foetal bovine serum (HI-FBS, Sigma) and 12.5 μg/mL G418 (Calbiochem) and 25 μg/mL hygromycin (Formedium) with 5% v/v CO_2_ atmosphere, as described. Cells were passaged every 2–3 days to maintain mid-log phase.

Epimastigote form *Trypanosoma cruzi* CL Brener strain were grown at 28°C in RTH medium [29] (RPMI 1640) (Sigma) supplemented with 20 mM HEPES pH 7.2, 4.9 mg/mL tryptone, PGAB (2 mM sodium glutamate, 2 mM sodium pyruvate, 100 μg/mL streptomycin and 100 U/mL penicillin, 20 mg/mL hemin and 10% HI-FBS; all Sigma). Cells were passaged every 3–4 days to maintain mid-log phase.

Promastigote form *Leishmania major* Freidlin strain were grown at 28°C in M199 medium (Sigma) pH 7.4, supplemented with 40 mM HEPES pH 7.4, 100 μM adenosine, 5 μg/mL hemin and 10% HI-FBS. Cells were passaged every 2–3 days to maintain mid-log phase.

HeLa cells (ATTC CCL-2) were grown at 37°C in Dulbecco’s Modified Eagle Medium (DMEM, Sigma) supplemented with 10% HI-FBS with 5% v/v CO_2_ atmosphere. Cells were passaged every 3–4 days to maintain confluence.

### Dose-response cytotoxicity assays

Specific details for each cell line are given below. In general: parasites were counted using CASY TT Cell Counter and mammalian cells were counted using a haemocytometer. Alamar blue cell viability assays were performed, whereby cells were incubated in the appropriate media containing the treatment compound (dissolved in DMSO) in serial dilution, after which the alamar blue cell viability reporter was added and the cells incubated further. Fluorescence was recorded using a FLx 800 plate reader (BioTek) with excitation wavelength 530–535 nm and emission wavelength at 590–610 nm using Gen5 Reader Control 2.0 Software (BioTek). EC_50_ values were determined using a 4-parameter non-linear logistic regression equation using GraFit 5.0 (Erithacus Software). SD values were calculated based on curve fitting to *n* biological replicates performed in parallel.

Bloodstream trypomastigote form *T*. *b*. *brucei* were maintained in HMI-11 medium as described above. Mid-log-phase cells were added to compound (2-fold serially diluted in HMI-11 growth medium) to a density of 1×10^3^ cells/well (final volume 200 μL) and incubated at 37°C with 5% v/v CO_2_ for 72 h. After which, 10 μL alamar blue (1.1 mg/mL resazurin sodium salt in PBS) was added to all wells and the cells incubated for a further 7 h before recording fluorescence.

Procyclic trypomastigote form *T*. *b*. *brucei* were maintained in SDM-79 medium as described above. Mid-log-phase cells were added to compound (2-fold serially diluted in SDM-79 growth medium) to a density of 1×10^4^ cells/well (final volume 200 μL) and incubated at 28°C with 5% v/v CO_2_ for 72 h. After which, 10 μL alamar blue (1.1 mg/mL resazurin sodium salt in PBS) was added to all wells and the cells incubated for a further 7 h before recording fluorescence.

Epimastigote *T*. *cruzi* CL Brener was maintained in RTH growth medium as described above. Mid-log-phase cells were added to compound (2-fold serially diluted in RTH growth medium) to a density of 5×10^5^ cells/well and incubated at 28°C (final volume 200 μL) for 72 h. After which, 10 μL alamar blue (1.1 mg/mL resazurin sodium salt in PBS) was added to all wells and the cells incubated for a further 7 h before recording fluorescence.

Promastigote *L*. *major* strain Freidlin were maintained in M199 growth medium as described above. Mid-log-phase cells were added to compound (2-fold serially diluted in M199 growth medium) to a density of 2×10^4^ cells/well (final volume 200 μL) and incubated at 28°C for 72 h. After which, 10 μL alamar blue (1.1 mg/mL resazurin sodium salt in PBS) was added to all wells and the cells incubated for a further 7 h before recording fluorescence.

HeLa cells were maintained in DMEM as described above. Cells were added to compound (2-fold serially diluted in HMI-11 growth medium) to a density of 1×10^4^ cells/well (final volume 200 μL) and incubated at 37°C with 5% CO_2_ for 72 h. After which, 10 μL alamar blue (1.1 mg/mL resazurin sodium salt in PBS) was added to all wells and the cells incubated for a further 7 h before recording fluorescence.

### Imaging of *T*. *brucei* cells

Bloodstream form *T*. *b*. *brucei* were grown in HMI-11 media containing EC_10_ concentration of the compound, or without the compound as a control, at 37°C with 5% CO_2_ for 72 h. Cells were counted and 1×10^6^ cells removed, adjusted to 1 mL final volume and Mitotracker red (Invitrogen) added to a final concentration of 100 nM. Samples were incubated for 30 min, 37°C, before cells were pelleted by centrifugation (1000 g, 10 min, 4°C) and the supernatant removed. Cells were washed with HMI-11 medium (1 mL), resuspended in fresh HMI-11 medium (1 mL) and incubated for 30 min, 37°C, before being pelleted by centrifugation (1000 g, 10 min, 4°C). Cells were fixed in paraformaldehyde solution (100 μL, 4% in PBS) and incubated in the dark for 10 min, 20°C. Cells were pelleted (1000 g, 10 min, 4°C), resuspended in PBS containing 1 μg/mL 4′,6-diamidino-2-phenylindole (DAPI, ThermoFisher) before mounting on slides. Cells were imaged using a DeltaVision Ultra microscope, measuring fluorescence independently for DAPI and MitoTracker red, as well as capturing differential image contrast (DIC) to show overall morphology. A total of 20 images were captured for both treated and untreated cells: 4 separate biological replicates, 5 images per replicate. The total number of clearly visible and non-overlapping parasites was 52 and 44 for untreated and treated conditions, respectively. Each image was captured on all 3 channels and processed using SoftWoRx imaging software. Representative images were selected for use in figures.

### Preparation of samples for proteomic analysis

Bloodstream form *T*. *b*. *brucei* were grown in HMI-11 media containing EC_10_ concentration of compound, or without the compound as a control, at 37°C with 5% CO_2_ for 72 h. Cells were counted and 2×10^7^ cells collected, then pelleted by centrifugation (1000 g, 10 min, 4°C) and the supernatant removed. Cells were resuspended in PBS (1.0 mL) and transferred to pre-cooled Eppendorf tubes, then pelleted by centrifugation (2500 g, 5 min, 4°C) and the supernatant removed. Cells were then washed with PBS (1.0 mL), pelleted by centrifugation (2500 g, 5 min, 4°C) and the supernatant removed. Cell pellets were lysed with aqueous urea (aq, 5 M, 20 μL) and stored at -80°C until analysis.

### MS analysis of cellular protein samples

Cell samples dissolved in urea (aq, 5 M, 20 μL) were analysed. Briefly, following disulfide reduction (dithiothreitol), cystine alkylation (iodoacetamide) and digestion by trypsin protease, peptide fragments were eluted through a C_18_ RP-LC column and analysed by MALDI-MS. Results were analysed by Mascot software compared to the *T*. *brucei* 927 database, both with and without a mass modification of tryptophan accounting for chlorination (+34 Da).

### Preparation of samples for metabolomics analysis

Cell and media metabolite extractions were performed in at least triplicate for each condition, using a single separate parasite culture for each replicate and compared to untreated control cell and media samples. Bloodstream form *T*. *b*. *brucei* were grown in HMI-11 media containing EC_10_ concentration of compound at 37°C with 5% CO_2_ for 72 h. Cells were counted and 1×10^8^ cells collected, then their metabolism was quenched by rapid cooling to 4°C using a CO_2_/ethanol bath. Cells were stored on ice for the remainder of the procedure. Cells were pelleted by centrifugation (1000 g, 10 min, 4°C) and 5 μL of the supernatant collected for metabolite analysis (spent media samples) with the remainder being discarded. Cells were resuspended in PBS (1.0 mL) and transferred to pre-cooled Eppendorf tubes, then pelleted by centrifugation (2500 g, 5 min, 4°C) and the supernatant removed. Cells were then washed with PBS (1.0 mL), pelleted by centrifugation (2500 g, 5 min, 4°C) and the supernatant removed. The cell pellets and spent media samples were resuspended in a cooled mixture of chloroform:methanol:water (1,3:1, 200 μL) followed by rocking (1 h, 4°C). Insoluble debris was then removed from the extraction mixture by centrifugation (13000 g, 5 min, 4°C) and the supernatant collected. A quality control sample was created to ensure accurate metabolite analysis by combining 20 μL of each sample within the study (both cells and media). Samples were stored under argon and frozen (-80°C) until analysis.

### MS analysis of metabolite samples

Metabolomics samples were analysed via liquid chromatography-mass spectrometry (LC-MS) based on methods previously described [30]. Hydrophilic interaction liquid chromatography (HILIC) was performed using a Dionex UltiMate 3000 RSLC system (Thermo Fisher Scientific, Hemel Hempstead, UK) using a ZIC-pHILIC column (150 mm x 4.6 mm, 5 μm column, Merck Sequant). The column was maintained at 25°C and samples were eluted with a linear gradient using the solvent system A) 20 mM (NH_4_)_2_CO_3_ in H_2_O and B) C_2_H_3_N over 26 minutes (flow rate 0.3 ml/min). Samples were injected (10 μl) and were maintained at 5°C prior to injection. MS analysis was performed using a Thermo Orbitrap QExactive (Thermo Fisher Scientific) operated in polarity switching mode (Resolution = 70000; AGC = 1e6; m/z = 70–1050; Sheath gas = 40, Auxiliary gas = 5, Sweep gas = 1; Probe temperature = 150°C, Capillary temperature = 320°C). For positive mode ionisation: source voltage +3.8 kV, S-Lens RF Level 30.00, S-Lens Voltage -25.00 (V), Skimmer Voltage -15.00 (V), Inject Flatopole Offset -8.00 (V), Bent Flatapole DC -6.00 (V). For negative mode ionisation: source voltage -3.8 kV. Pooled samples were employed for quality control purposes to assess reproducibility of the instrument, being analysed at 5-sample intervals throughout the run. Data analysis was performed using the IDEOM analysis pipeline [31] for data filtering and metabolite annotation. Metabolite annotations are assigned at Level 2 in accordance with Metabolomics Standards Initiative directives [32], given annotations are putative annotations made based on accurate masses and predicted retention times. For metabolites matched to authentic standards in IDEOM, putative annotations equate to Level 1 identification. IDEOM Excel output files and raw data files are available from the Metabolights database [33] (https://www.ebi.ac.uk/metabolights/MTBLS1657). The Polyomics Integrated Metabolomics Pipeline (PiMP)[34] was also used for data visualisation, particularly for the assessment of fragmentation data.

### Synthesis of compounds 8–13

Parent halogenated l-tryptophan derivatives **1**–**7** were kindly synthesized and provided by R. J. M. Goss and co-workers (University of St Andrews) in their free-base form and were used without further purification. Information on their synthesis and characterization has been presented in a recent publication [35].

General Procedure for the Preparation of Substituted l-Tryptophan Methyl Esters **8–13**

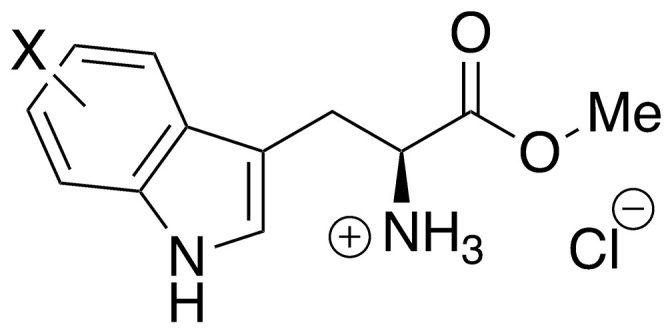


To a solution of substituted l-tryptophan (ca. 45 mg) in methanol (2 mL) at 0°C was added Acetyl chloride (200 μL) dropwise. The reaction was heated to reflux for 2 h then the solvents removed under reduced pressure to give substituted tryptophan methyl esters as their hydrochloride salts which were used without further purification.

**8** –methyl 5-chloro-l-tryptophanate hydrochloride

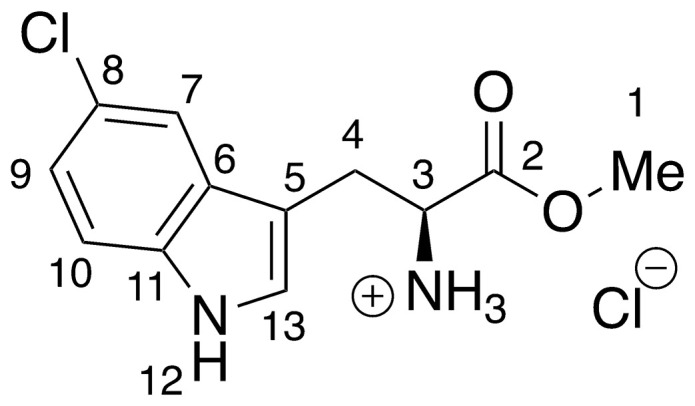


**8** was prepared *via* the general method above, using 5-chloro-l-tryptophan (46.1 mg, 0.19 mmols) to give the ester as an off white solid (54.1 mg, 97%). ^**1**^**H NMR** (500 MHz, MeOD) δ 7.51 (d, *J* = 1.9 Hz, 1H, H_7_), 7.35 (d, *J* = 8.6 Hz, 1H, H_10_), 7.26 (s, 1H, H_13_), 7.09 (dd, *J* = 2.0, 8.6 Hz, 1H, H_9_), 4.31 (t, *J* = 6.2 Hz, 1H, H_3_), 3.79 (s, 3H, H_1_), 3.45–3.31 (m, 2H, H_4_).

**9** –methyl 6-chloro-l-tryptophanoate hydrochloride

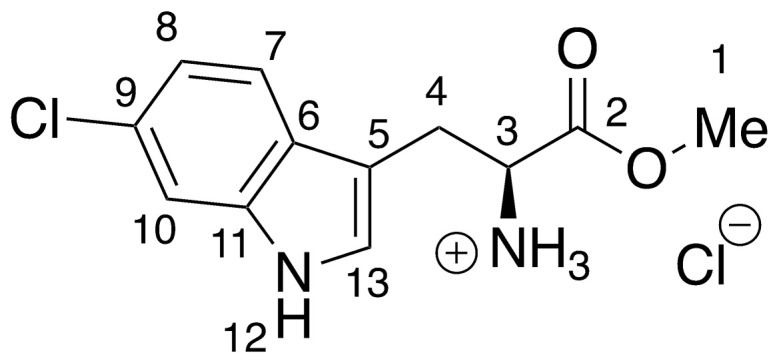


**9** was prepared *via* the general method above, using 6-chloro-l-tryptophan (46.6 mg, 0.20 mmols) to give the ester as a pale brown solid (56.0 mg, >99%). ^**1**^**H NMR** (500 MHz, MeOD) δ 7.52 (d, *J* = 8.4 Hz, 1H, H_7_), 7.42 (d, *J* = 1.7 Hz, 1H, H_10_), 7.26 (s, 1H, H_13_), 7.06 (dd, *J* = 1.7, 8.5 Hz, 1H, H_8_), 4.35 (t, *J* = 6.2 Hz, 1H, H_3_), 3.80 (s, 3H, H_1_), 3.48–3.38 (m, 2H, H_4_).

**10** –methyl 7-chloro-l-tryptophanoate hydrochloride

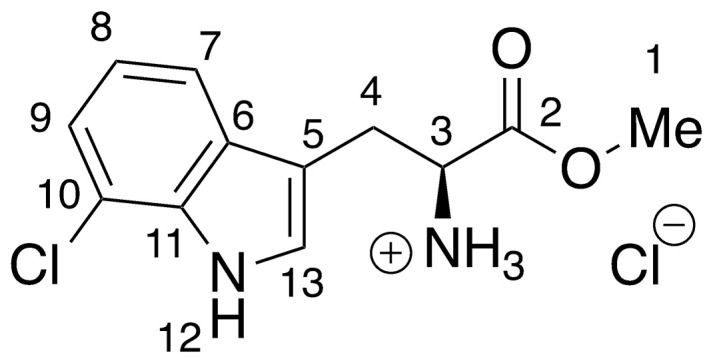


**10** was prepared *via* the general method above, using 7-chloro-l-tryptophan (45.7 mg, 0.19 mmols) to give the ester as a cream solid (55.2 mg, >99%). ^**1**^**H NMR** (500 MHz, MeOD) δ 7.52 (dd, *J* = 0.9, 7.9 Hz, 1H, H_7_), 7.32 (s, 1H, H_13_), 7.19 (dd, *J* = 0.8, 7.6 Hz, 1H, H_9_), 7.07 (t, *J* = 7.8 Hz, 1H, H_8_), 4.36 (dd, *J* = 5.7, 7.1 Hz, 1H, H_3_), 3.80 (s, 3H, H_1_), 3.54–3.37 (m, 2H, H_4_).

**11** –methyl 5-bromo-l-tryptophanoate hydrochloride

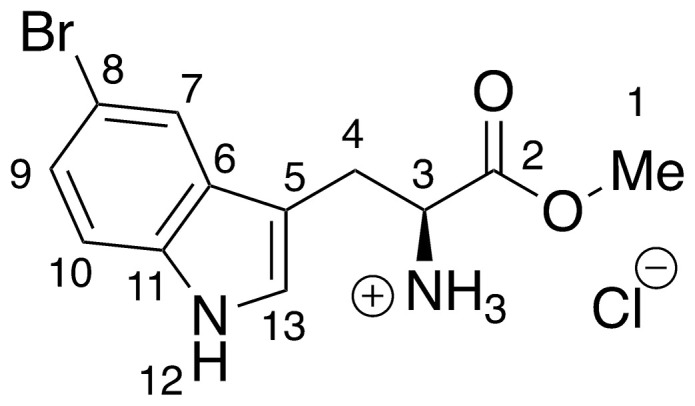


**11** was prepared *via* the general method above, using 5-bromo-l-tryptophan (43.9 mg, 0.16 mmols) to give the ester as a red-brown solid (48.0 mg, 98%). ^**1**^**H NMR** (500 MHz, MeOD) δ 7.70 (d, J = 1.8 Hz, 1H, H_7_), 7.35 (d, J = 8.6 Hz, 1H, H_10_), 7.27 (s, 1H, H_13_), 7.25 (dd, J = 1.8, 8.6 Hz, 1H, H_9_), 4.35 (t, J = 6.2 Hz, 1H, H_3_), 3.83 (s, 3H, H_1_), 3.40 (t, J = 6.4 Hz, 2H, H_4_).

**12** –methyl 6-bromo- l-tryptophanoate hydrochloride

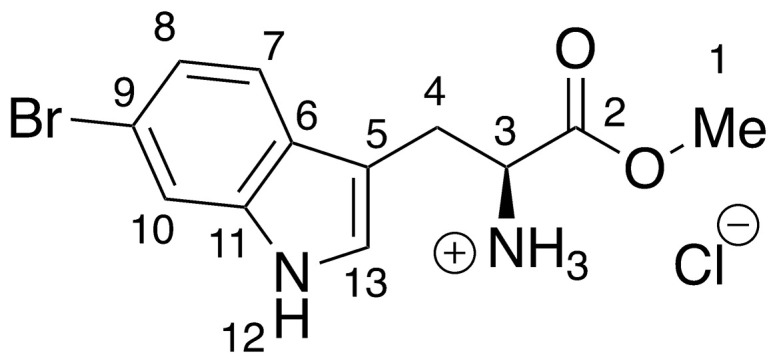


**12** was prepared *via* the general method above, using 6-bromo-l-tryptophan (43.0 mg, 0.15 mmols) to give the ester as a pale brown solid (47.9 mg, >99%). ^**1**^**H NMR** (500 MHz, MeOD) δ 7.59 (d, J = 1.7 Hz, 1H, H_10_), 7.48 (d, J = 8.5 Hz, 1H, H_7_), 7.25 (s, 1H, H_13_), 7.20 (dd, J = 1.7, 8.5 Hz, 1H, H_8_), 4.35 (t, J = 6.4 Hz, 1H, H_3_), 3.81 (s, 3H, H_1_), 3.45 (dd, J = 5.6, 15.2 Hz, 1H, H_4_), 3.38 (dd, J = 7.1, 15.3 Hz, 1H, H_4’_).

**13** –methyl 7-bromo- l-tryptophanoate hydrochloride

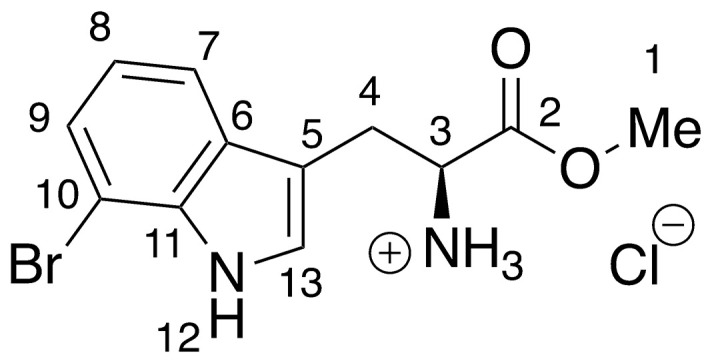


**13** was prepared *via* the general method above, using 7-bromo-l-tryptophan (47.6 mg, 0.17 mmols) to give the ester as a red-brown solid (53.1 mg, >99%).^**1**^**H NMR** (500 MHz, MeOD) δ 7.56 (d, J = 7.9 Hz, 1H, H_7_), 7.34 (d, J = 7.5 Hz, 1H, H_9_), 7.34 (s, 1H, H_13_), 7.02 (t, J = 7.8 Hz, 1H, H_8_), 4.36 (t, J = 6.4 Hz, 1H, H_3_), 3.80 (s, 3H, H_1_), 3.47 (dd, J = 5.7, 15.2 Hz, 1H, H_4_), 3.40 (dd, J = 7.1, 15.2 Hz, 1H, H_7_).

**14** –methyl 7-iodo- l-tryptophanoate hydrochloride

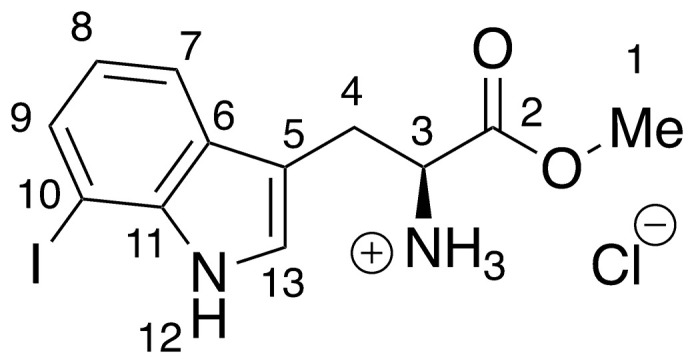


**14** was prepared *via* the general method above, using 7-iodo-l-tryptophan (49.2 mg, 0.15 mmols) to give the ester as a cream solid (51.4 mg, 90%). ^**1**^**H NMR** (400 MHz, MeOD) δ 7.58 (dd, *J* = 0.9, 8.0 Hz, 1H, H_7_), 7.56 (dd, *J* = 0.9, 7.5 Hz, 1H, H_9_), 7.32 (s, 1H, H_13_), 6.89 (t, *J* = 7.7 Hz, 1H, H_8_), 4.36 (dd, *J* = 5.7, 7.1 Hz, 1H, H_3_), 3.80 (s, 3H, H_1_), 3.50–3.34 (m, 2H, H_4_).

## Results

### Synthesis and initial bioactivity

**Fig 1:**
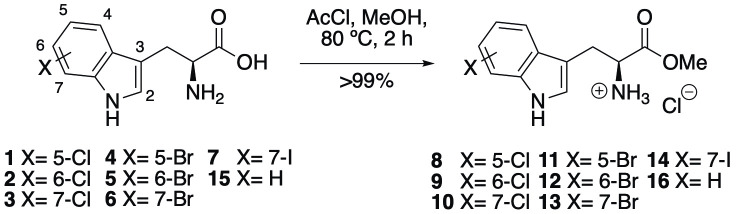
Conversion of halogenated tryptophan analogues 1–7 to their corresponding methyl esters 8–14, as well as natural tryptophan (15) to 16. The general structure for tryptophan analogues **1**–**7** is labelled with standard indole ring system numbering for clarity.

Recent work by Goss and co-workers investigated modified tryptophan synthase enzymes capable of synthesizing a range of halogenated tryptophan derivatives from halo-indole and serine [35]. A range of substituted tryptophan analogues (**1–7**) were chosen for investigation into their trypanocidal activities. Free acids **1–7** were also converted to their corresponding methyl esters **8–14**, in order to aid cellular uptake by masking of the carboxylate functionality (Fig 1). Compounds **1–14** were tested for bioactivity activity against *T*. *brucei* bloodstream form (BSF) trypomastigotes and the HeLa mammalian cell line, alongside natural tryptophan (**15**) and its methyl ester (**16**). Corresponding EC_50_ values and selectivity indexes (SI) are shown in Table 1. Compounds were also tested against *Trypanosoma cruzi* epimastigotes and *Leishmania major* amastigotes, however, they showed no activity below 250 μM (S1 Table).

**Table 1:** Bioactivity of tryptophan derivatives 1–7 and their methyl esters 8–14, as well as natural tryptophan (15) and its methyl ester 16, against *T*. *brucei* bloodstream form trypomastigotes and HeLa cells. Mean half maximal growth inhibition (EC_50_) values (μM) determined from *n* = 4 replicates, all SD values (not shown) were within 10% of each EC_50_ value. nd-not determined.

**Free Acids**	**1**	**2**	**3**	**4**	**5**	**6**	**7**	**15**
*T*. *brucei*^a^ (SI^d^)	>250 (nd)	>250 (nd)	47.4 (>5.3)	>250 (nd)	>250 (nd)	46.1 (>5.4)	42.0 (>5.9)	>250 (nd)
HeLa	>250	>250	>250	>250	>250	>250	>250	>250
**Methyl Esters**	**8**	**9**	**10**	**11**	**12**	**13**	**14**	**16**
*T*. *brucei*^a^ (SI^d^)	149.5 (>1.7)	157.9 (>1.6)	3.5 (>71)	101.7 (>2.5)	139.7 (>1.8)	3.7 (>67)	2.7 (>92)	>250 (nd)
HeLa	>250	>250	>250	>250	>250	>250	>250	>250

^a^ *Trypanosoma brucei brucei* bloodstream form trypomastigotes.

^d^EC50 of mammalian cells/EC50 off parasite

A distinct trend in bioactivity was observed regarding the different positions of indole halogenation, where 7-substituted compounds displayed significantly more potent activity than their 5 and 6-substituted counterparts. The trend was conserved for both compound types, with free acids **3**, **6**, **7** and methyl esters **10**, **13**, **14** displaying up to 45-fold higher trypanocidal activity than their analogues bearing alternative substitution patterns. Methyl esterification increased trypanocidal activity of all compounds except natural L-tryptophan, with 7-substituted compounds **10**, **13** and **14** displaying a >10-fold increase over **3**, **6** and **7**, respectively. All compounds showed no detectable activity against the HeLa human cell line, giving compounds **10**, **13** and **14** promising selectivity indexes (SI) of 71.4, 67.6, 92.6, respectively. This data provided encouraging evidence that these compounds possess a trypanosome-selective mechanism of cytotoxicity.

*T*. *brucei* express prolific cytosolic esterase enzymes, which are capable of hydrolyzing a broad range of esters. It is likely that esters **7–14** are precursors that are rapidly cleaved within the parasite cells, giving the corresponding free acids as the active intracellular agents. The improved activity of methyl esters **7–14** can, therefore, be explained by their improved cell permeability, due to their ability to passively diffuse across trypanosomal membranes [36]. Their free acid counterparts **1–7** would likely require the use of aromatic amino acid transporters to permeate cells [13,37] and would, therefore, display comparatively reduced bioavailability. It is not clear whether methyl esters may also capitalize on the use of these transporters to gain additional cellular permeability.

**Fig 2:**
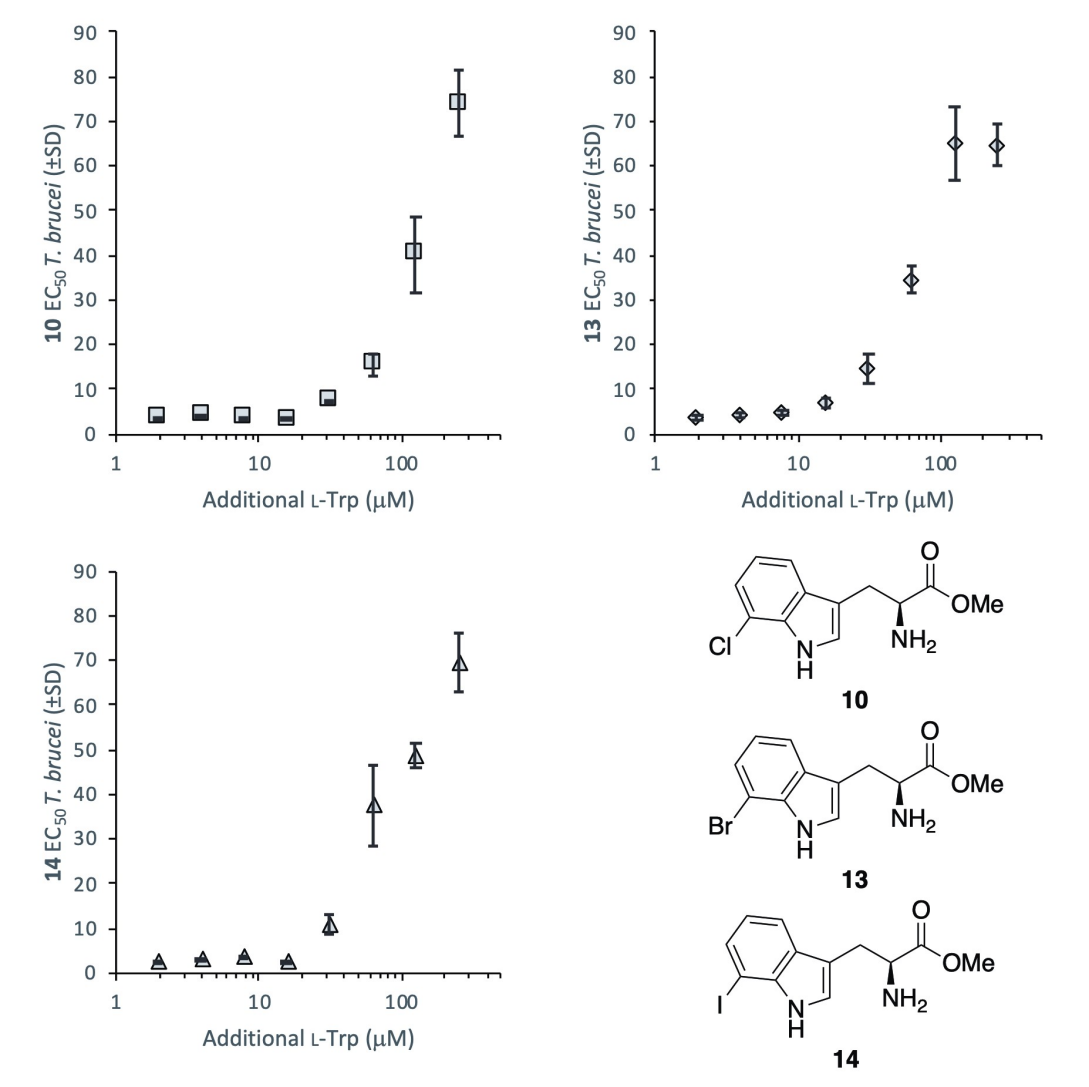
The effect of supplementary tryptophan (μM) added to media on the EC_50_ values (μM ±SD) of substituted tryptophan methyl esters 10, 13 and 14 against *T*. *brucei* BSF. SD determined from *n* = 3 replicates.

As closely related structural analogues of tryptophan, we first sought to investigate the relationship between analogue activity against *T*. *brucei* and concentrations of natural tryptophan within growth medium. The EC_50_ values of the most active methyl esters **10**, **13** and **14** were measured in varying levels of tryptophan supplementation (Fig 2), revealing a decrease in compound potency when exposed to elevated levels of tryptophan (>15 μM supplement). In growth medium treated with the maximal 250 μM of additional tryptophan, compounds displayed approximately 20-fold reduced activity.

### Proteomic analysis

Since tryptophan is an important amino acid in protein structure [38], we assessed the potential incorporation of substituted tryptophan analogues into trypanosomal protein synthesis, which could potentially result in the production of non-functional proteins. Cellular protein samples from *T*. *brucei* treated with 3 μM (EC_10_) of the 7-chloro methyl ester **10** were digested with trypsin and analyzed by mass spectrometry (MS). Analysis of the resulting MS Data by MASCOT identified 5 peptides in treated parasites potentially containing the +34 Da modification (corresponding to the substitution of Trp with ^35^Cl-Trp), and 1 peptide in untreated parasites (S2 Table and S3 Table). Each of these six peptides belonged to a separate protein and only the modified peptide was detected for each protein. Assignments are, therefore, of low confidence, meaning identification of these modified peptide masses may be a result of mis-assignment. As incorporation into protein synthesis would be ubiquitous, the absence of Cl-Trp in high-abundance, high-turnover proteins (such as tubulin α and β subunits), as well as the low confidence in assigning possible detected peptides, suggests that the 7-substituted tryptophan analogues are not incorporated during protein synthesis.

### Metabolomic analysis

**Fig 3:**
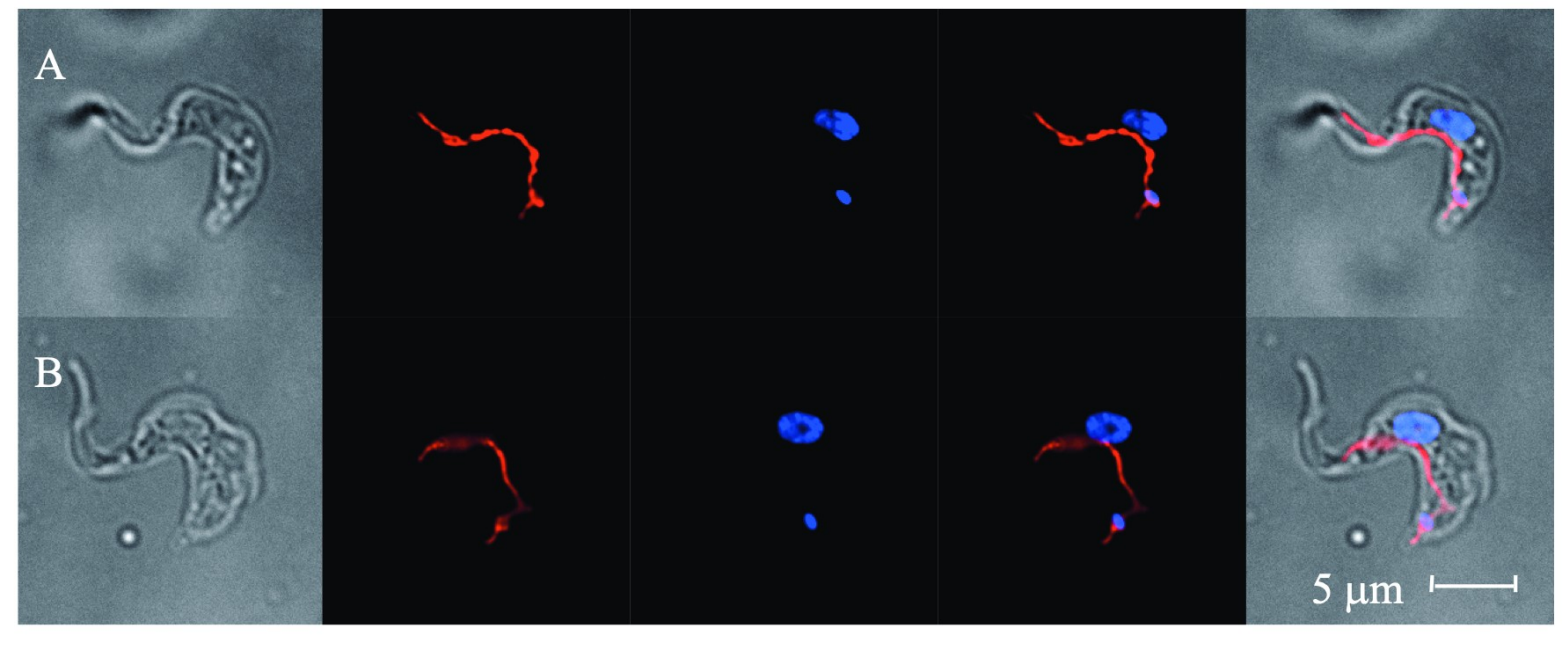
Representative images of *T*. *brucei* BSF. **A.** Control cells. **B.** Cells treated with analogue **10** (EC_10_). From left to right: DIC microscopy identifying cellular structure; MitoTracker Red fluorescence identifying parasite mitochondria; DAPI fluorescence identifying parasite DNA (nucleus and kinetoplast); DAPI and MitoTracker Red fluorescence overlay; DIC, DAPI and MitoTracker Red overlay.

We next investigated the effect of the substituted tryptophan analogues on the amino acid metabolism of *T*. *brucei*. Trypanosomes grown in both treated (with EC_10_ of ester **10** for 72 hr) and untreated media were stained with DAPI and MitoTracker Red, to highlight nuclear and mitochondrial structures, then examined by optical and fluorescence microscopy (Fig 3). Parasites grown in both treated and untreated media show healthy morphology, with preserved mitochondrial form and function (shown by the successful accumulation of mitotracker dye, which requires mitochondrial potential, (Fig 3). K/N analysis was performed, albeit with a somewhat low sample size (n = 52 and 44 for untreated and treated, respectively), and showed comparable values between treated and untreated parasites: Untreated 1K/1N 67%; 1K*/1N 19%; 1K/1N* 4%; 2K/1N 4%; 2K/2N 2%. Treated 1K/1N 61%; 1K*/1N 18%; 1K/1N* 7%; 2K/1N 11%; 2K/2N 2%. (* denotes an elongated organelle, likely in the process of replication). The maintenance of healthy cellular function was crucial to subsequent metabolomic investigations, so that observations could be linked solely to analogue effect and not metabolic shutdown caused by cell death.

Due to the limited supply of the tryptophan analogues, the 7-bromo ester **13** was used for further metabolomic analysis. Since the compounds’ structures and efficacy are highly similar, it is likely that these compounds’ metabolic effects are also comparable. Parasites were grown in media treated with the 7-bromo ester **13** to a final concentration of 3 μM (EC_10_) and 7 μM (EC_30_), then cellular metabolites were extracted for analysis, along with metabolites extracted from the spent media of EC_10_ treated parasites. Cellular and media metabolites were extracted from parasites grown in untreated media, as well as naïve media and analyzed as control samples. Extracted metabolites were analyzed by ZIC-pHILIC LC-MS and spectra were processed using IDEOM [31] as a platform for peak identification and metabolite analysis. As an untargeted metabolomics approach, calculation of compound or metabolite concentrations is beyond the scope of the employed methods. Instead, relative levels of analyte detection by MS (ion counts) are used to compare metabolite amounts within each sample and, as such, data are qualitative in nature and should not be considered as absolute concentrations. It should be noted untreated parasite samples display a wider spread than treated samples. One control replicate consistently displayed slightly lower metabolite counts than the remaining two, however the sample was included in analyses to show a true reflection of the complete dataset.

**Fig 4:**
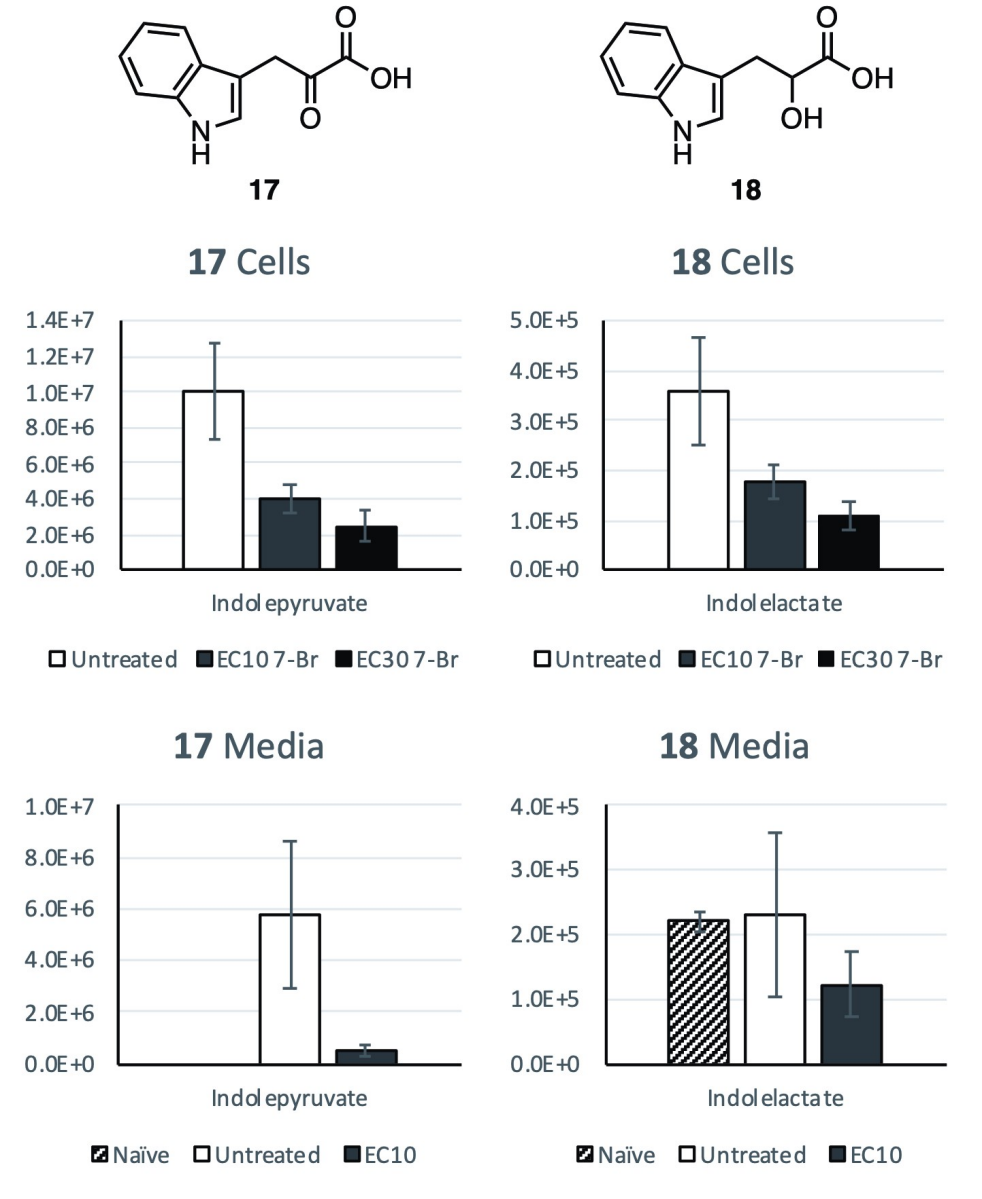
The effect of 7-Br tryptophan methyl ester (13) treatment on the relative medium and cellular levels of selected metabolites indolepyruvate (17) and indole lactate (18). Relative levels are detected ion counts by MS from separate samples. Error bars show SD calculated from *n* = 3 replicates.

The initial focus was directed towards metabolites directly involved with tryptophan metabolism in *T*. *brucei*. This was prompted by the close structural homology between tryptophan and the treatment compound **13** (as well as active analogues **10** and **14**) and the observed disruption of **10**, **13** and **14**’s trypanocidal activity upon addition of excess tryptophan, which indicated direct competition. Indolepyruvate (**17**), the major reported product of tryptophan metabolism by *T*. *brucei*, was observed in significantly reduced levels in both EC_10_ and EC_30_ treated cell extracts (61% and 75% reduction, respectively) compared to untreated cells. **17** was also greatly reduced (91% reduction) in spent EC_10_ treated media, compared to spent untreated media (Fig 4). Levels of the downstream metabolite indole lactate (**18**) were similarly reduced in treated cells, although showed a less significant reduction in spent media than **17**, likely due to the presence of **18** in naïve media. Alternatively, indole lactate (18) may be consumed by the cells when treated 13 even at its EC10.

**Fig 5:**
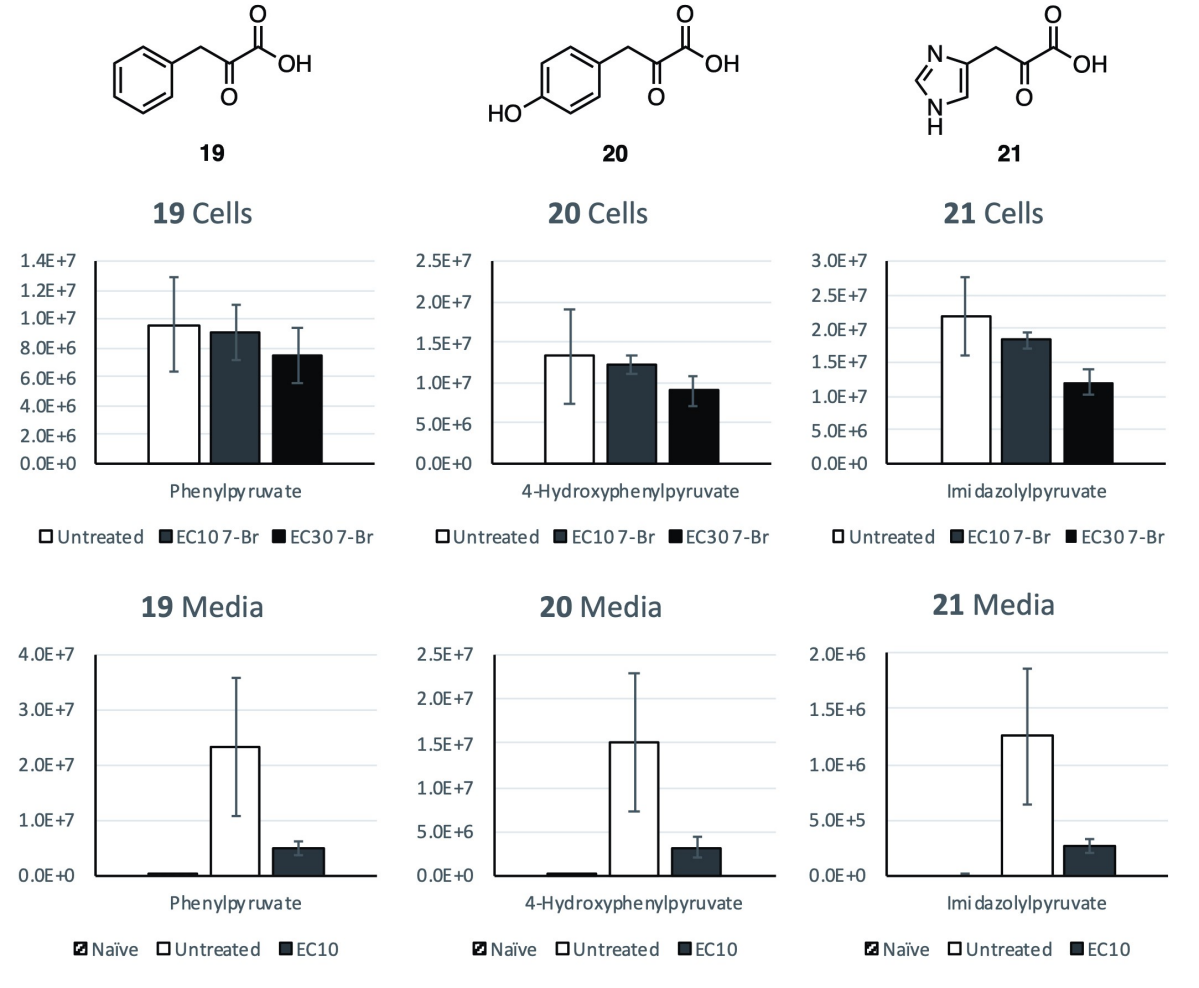
The effect of 7-Br tryptophan methyl ester (13) treatment on the relative medium and cellular levels of selected metabolites phenylpyruvate (19), 4-hydroxyphenylpyruvate (20), and imidazolylpyruvate (21). Relative levels are detected ion counts by MS from separate samples. Error bars show SD calculated from *n* = 3 replicates.

The related metabolites phenylpyruvate (**19**), 4-hydroxyphenylpyruvate (**20**) and imidazolylpyruvate (**21**), the transamination products of the aromatic amino acids phenylalanine, tyrosine and histidine, are also widely reported cellular products of *T*. *brucei* [19]. Levels of **19**, **20** and **21** were significantly reduced in treated media, with a 79% decrease in detected levels for all three annotated metabolites. Cellular levels of these metabolites were comparatively better maintained in treated parasites: **19** showed 5% for EC_10_ and 22% for EC_30_ treated cells; **20** showed 8% for EC_10_ and 32% for EC_30_ treated cells; while **21** showed 13% for EC_10_ and 45% for EC_30_ treated cells, respectively (Fig 5).

We next searched the recorded metabolite profile for metabolite masses accounting for compound **13**, as well as any potential brominated analogues of reported tryptophan metabolites. The parent compound **13** could not be detected in significant levels in either treated cell (EC_10_ or EC_30_) or media (EC_10_) media extracts. The brominated free acid **6** was detected in small amounts in cells grown in EC_30_ of compound **13**, however, this had been consumed beyond detectable levels in cells grown in EC_10_ concentration. Two distinct brominated metabolites with equivalent molecular masses of 280.9688 {^79^Br} and 282.9669 {^81^Br} were identified in treated cell extracts (EC_10_ and EC_30_). The first metabolite was identified as the transamination product 7-bromoindolepyruvate (**22**), through comparison of retention time to natural indole pyruvate (**17**) (RT_**22**_ = 7.07; RT_**23**_ = 7.03–7.02). The second metabolite (referred to as compound **23**) could not be aligned to a known natural tryptophan metabolite of similar retention time (RT_**24**_ = 6.19–6.24), so a probable structure could not be assigned. **23** is likely a specific product of compound **13**’s metabolism, or a typically short-lived intermediate, which is stabilized within the cell by the bromo-modification. No brominated metabolites were detected in significant levels within media extracts.

**Fig 6:**
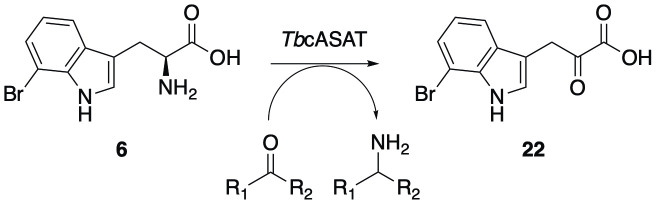
Proposed turnover of substituted tryptophan 6 to its corresponding α-ketoacid 22 by TbcASAT.

The absence of **13** in media or cellular samples confirms the uptake and rapid hydrolysis of the analogue by *T*. *brucei*, confirming its status as a precursor of the free acid **6**, which is subject to further metabolism within parasites. It is clear that the modified tryptophan **6** is a suitable substrate for the well-reported prolific transamination of natural tryptophan by *T*. *brucei* and is itself converted into the brominated pyruvate derivative **22** (Fig 6).

Conversion of the treatment analogue, **13** first to the parent tryptophan analogue **6**, then to 7-bromoindolepyruvate (**22**), combined with the observed changes in the key keto-acid metabolites **17** and **19**–**21** may allude to its trypanocidal mechanism of action. The transamination of tryptophan, phenylalanine and tyrosine within *T*. *brucei* BSF is catalyzed by a cytosolic aspartate aminotransferase (TbcASAT) [26,39,40]. The transamination products **17**, **19** and **20** were all observed at significantly decreased levels in the spent media of treated cells with average reductions of 91%, 79% and 79%, respectively, compared to untreated samples. These observations point to the significantly reduced activity of TbcASAT within treated parasites, and are in-line with similar reductions in overall aromatic keto-acid production from induced RNAi cASAT cells measured by Nolan and co-workers [26]. These findings suggest that **13**, or one of its metabolites **6** and **22**, acts as a potential inhibitor of TbcASAT. Although the enzyme responsible for the transamination of histidine in *T*. *brucei* has not been fully characterized, the cytosolic fraction of *T*. *brucei* has been shown to successfully complete this process [39]. We suggest that TbcASAT may also be responsible for this transamination process, explaining the similar observed decrease in the production of **21** within treated cells.

**Fig 7:**
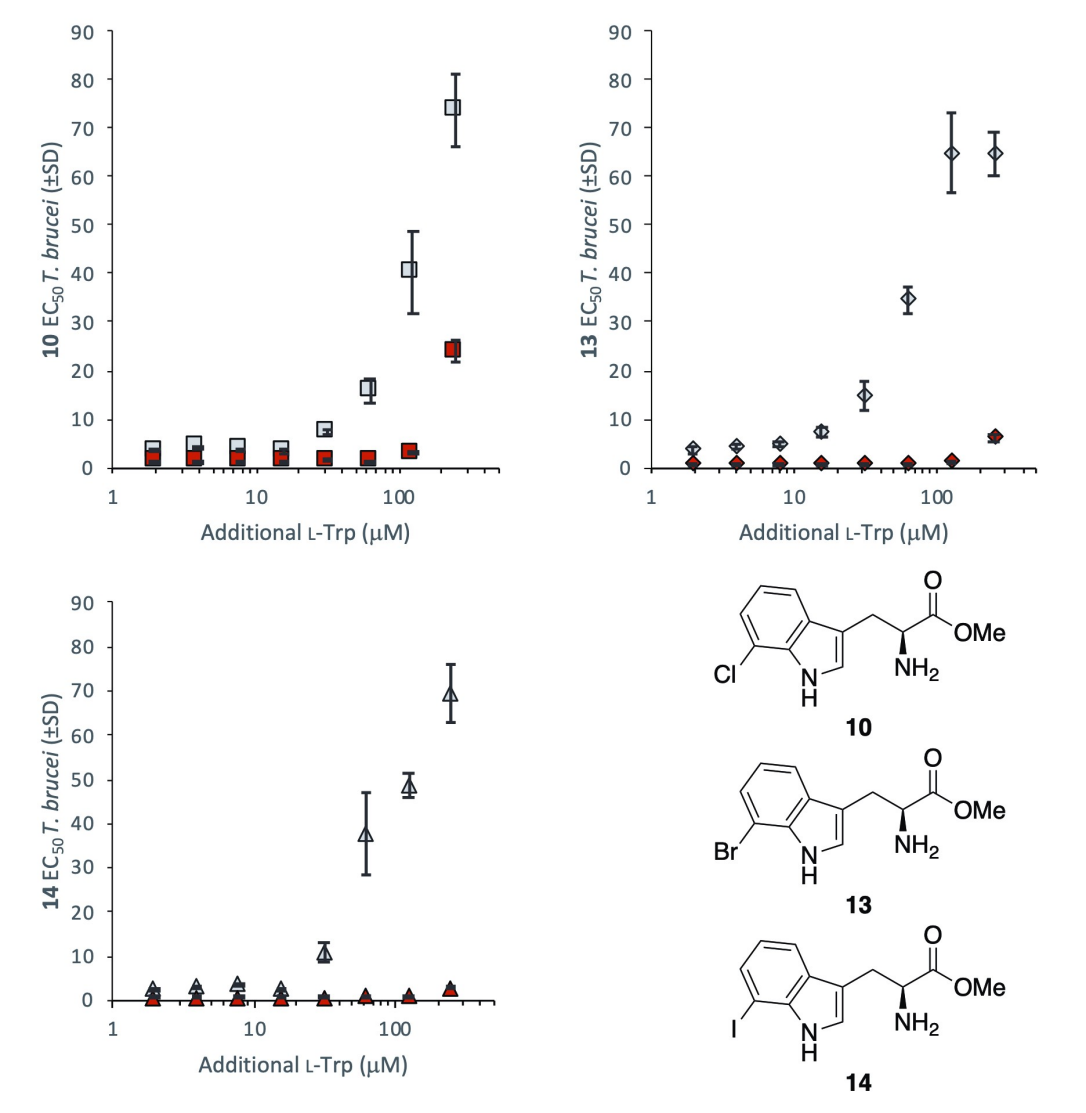
The effect of supplementary tryptophan (μM ±SD) added to media on the EC_50_ values (μM ±SD) of substituted tryptophan methyl esters 10, 13 and 14 against *T*. *brucei* BSF (grey) and *T*. *brucei* PCF (red) parasites. SD determined from *n* = 3 replicates.

TbcASAT has been shown to be essential in *T*. *brucei* BSF [26], thus is a reasonable candidate for the trypanocidal target of the compound series. Since mammalian cells do not engage in the rapid transamination of these aromatic amino acids this, importantly, provides a mechanism for the observed selective trypanosomal growth inhibition. In the procyclic form (PCF) of *T*. *brucei*, a highly specific mitochondrial aspartate aminotransferase (TbmASAT) accounts for transamination activity using only aspartate, with TbcASAT comprising a minor portion of ASAT activity. [26,40] The activity of tryptophan derivatives **1**–**16** were examined against PCF *T*. *brucei*, revealing a similar bioactivity profile to that observed in BSF *T*. *brucei*, with 7-substituted esters **10**, **12** and **13** displaying the most potent activity against both parasite forms (Table 2). Notably, however, the addition of natural l-tryptophan did not diminish compound activity against PCF *T*. *brucei* as drastically as was seen with BSF. Compound potency was modified only at the maximum 250 μM supplement level (Fig 7), significantly higher than the 15 μM tryptophan supplement that caused a reduction in activity within BSF *T*. *brucei*.

**Table 2:** Bioactivity of tryptophan derivatives 1–7 and their methyl esters 8–14, as well as natural tryptophan (15) and its methyl ester 16, against *T*. *brucei* bloodstream form trypomastigotes (BSF) and *T*. *brucei* procyclic form trypomastigotes (PCF). Mean EC_50_ values (μM) determined from *n* = 4 replicates, all SD values (not shown) within 10% of EC_50_ value.

**Free Acids**	**1**	**2**	**3**	**4**	**5**	**6**	**7**	**15**
*T*. *brucei* BSF^a^	>250	>250	47.4	>250	>250	46.1	42.0	>250
*T*. *brucei* PCF^b^	>250	>250	39.7	>250	154.2	33.8	47.6	>250
**Methyl Esters**	**8**	**9**	**10**	**11**	**12**	**13**	**14**	**16**
*T*. *brucei* BSF^a^	149.5	157.9	3.5	101.7	139.7	3.7	2.7	>250
*T*. *brucei* PCF^b^	82.8	81.2	3.1	77.2	72.4	1.8	1.5	>250

^a^ *Trypanosoma brucei brucei* bloodstream form trypomastigotes.

^b^ *Trypanosoma brucei brucei* procyclic form trypomastigotes.

## Discussion

The substituted tryptophan analogues **1**–**14** show a range of trypanocidal activity against *T*. *brucei* BSF, with 7-substituted methyl ester analogues **10**, **13** and **14** showing low-micromolar activity and high selectivity. Compounds **10**, **13** and **14** display a competitive relationship with l-tryptophan, which diminishes their trypanocidal activity at elevated levels in growth media. Since tryptophan is actively taken up by *T*. *brucei*, it is possible that methyl esters **10**, **13** and **14** compete with tryptophan for active transport, as well as passively diffusing across the cellular membrane. Similar studies have shown that modified sugar derivatives, including halogenated examples, exhibit trypanocidal activity through the inhibition of key hexose transporters. [41]

The metabolomics data indicate that ester **13** is rapidly cleaved within the cytosol of *T*. *brucei* to give free acid **6**, then converted to the corresponding substituted pyruvate **22**, most likely catalyzed by the aminotransferase TbcASAT. The rapid hydrolysis of ester **13** means it is unlikely to be the active trypanocidal agent, however the ester is obviously acting as a pro-drug and therefore either the free acid or a downstream metabolite thereof is the active trypanocidal compound. Reduced excretion of aromatic α-ketoacids indolepyruvate, phenylpyruvate and 4-hydroxyphenylpyruvate suggest reduced activity of TbcASAT in treated parasites. Since TbcASAT is not essential within PCF *T*. *brucei*, its inhibition is not likely to account for the trypanocidal activity observed in this parasite form. The main aminotransferase of PCF *T*. *brucei* is a highly aspartate-specific enzyme mASAT, which is unlikely to bind tryptophan analogues. TbcASAT is still expressed in PCF *T*. *brucei* parasites, albeit with lower activity, meaning it is possible that 7-substituted compounds **10**, **13** and **14** are similarly converted to their corresponding indolepyruvate derivatives within this parasite form. This could implicate halogenated indolepyruvate derivatives (i.e. **22** in the context of treatment with **13**) or other downstream metabolites as the active antiparasitic agents. The significant differences between the l-tryptophan competition profile of compounds **10**, **13** and **14** within BSF and PCF parasites suggest differences in the compounds’ mechanism of action. This may, however, be an observed effect of morphological differences between PCF and BSF parasites, such as the reduced cASAT activity [26] or differences in amino acid uptake and metabolism [13]. The metabolite profiles of PCF *T*. *brucei* treated with **13** have not been recorded, so it is unclear whether any downstream metabolites of **13** are produced within PCF parasites, making it challenging to comment upon a potential shared trypanocidal mode of action.

The failure to identify any brominated tryptophan metabolites in treated media samples, despite their production in significant quantities within the cells, suggests that brominated analogues are not exported. The close structural similarity between brominated and natural analogues may enable the brominated metabolites **6** or **22** to bind and inhibit transporters responsible for the efflux of natural substrates. Intracellular accumulation of these brominated metabolites may slow efflux of normal metabolites, leading to the observed toxic accumulation of natural and unnatural metabolites and cell death. This mechanism of activity could also explain the compounds’ activity against PCF *T*. *brucei*, which retain the capability to transaminate **13**. with inhibition of efflux causing general shutdown of key cellular processes.

The trypanocidal activity of the treatment compound **13** may result from either one or, indeed, a combination of both of these mechanisms, with both inhibiting key metabolic processes. Furthermore, the unknown brominated metabolite **23** could contribute to the trypanocidal activity of the treatment compound **13** through either of the two discussed potential mechanisms, or through a separate mechanism. Its structure, unfortunately, could not be assigned by analogy to natural metabolites, and further analysis is required to determine its importance in the observed metabolic effects. The metabolic evidence presented, showing a clear reduction in key transamination products, supports the inhibition of TbcASAT in the trypanocidal mechanism of **13** within BSF *T*. *brucei*. It is unclear whether **13** or its downstream metabolites **6**, **22** or **23** are the true trypanocidal agents in this mechanism of action.

Key future experiments could further examine the trypanocidal mechanism of action of the 7-substituted tryptophan derivatives. *In vitro* examination of these compounds against purified TbcASAT activity could dissect whether the enzyme’s inhibition is involved in the trypanocidal action. In such experiments, tryptophan derivative **9**, methyl ester **13**, keto-acid **22** and the unknown metabolite **23** (following structural determination) should be included to identify the trypanocidal agent. A second metabolomics study could be conducted to include PCF *T*. *brucei* and targeted to specifically investigate and quantify transamination processes. Additionally, examination of the trypanocidal compound **13** *in vivo* using animal models could reveal therapeutic benefits of disrupting transamination processes in *T*. *brucei* infection. Examples proposed in the literature include combatting trypanosomal evasion of the host immune system [26], as well as alleviating late-stage symptoms that may be caused by associated products [21].

Despite the absence of an unequivocally confirmed trypanocidal target, the 7-substituted esters **10**, **13** and **14** display potent trypanocidal activity and a clear disruption of the widely reported transamination processes within *T*. *brucei*. These compounds may be used to aid future studies into the functional significance of transamination processes within *T*. *brucei* in both *in vitro* and *in vivo* systems, and in the future develop new anti-trypanosomal therapies targeting these important systems.

## Supporting information

S1 Table Bioactivity of tryptophan derivatives 1–7 and their methyl esters 8–14, as well as natural tryptophan (15) and its methyl ester 16, against T. brucei bloodstream form trypomastigotes, T. brucei procyclic form trypomastigotes, T. cruzi epimastigotes, L. Major promastigotes, and HeLa cells. Mean half maximal growth inhibition (EC_50_) values ±SD (μM) determined from n = 4 replicates.
(DOCX)

S2 Table Proteomic analyses of control T. brucei BSF parasites (no drug). Proteins are ranked by MASCOT assigned protein score, with the top ten results being shown in black, and proteins with Cl-Trp modified peptides detected shown in red. Protein scores are derived from ions scores (−10log[P], where P is the probability that the observed match is a random event) as a non-probabilistic basis for ranking protein families.
(DOCX)

S3 Table Proteomic analyses of T. brucei BSF parasites treated with tryptophan methyl ester analogue 10. Proteins are ranked by MASCOT assigned protein score, with the top ten results being shown in black, and proteins with Cl-Trp modified peptides detected shown in red. Protein scores are derived from ions scores (−10log[P], where P is the probability that the observed match is a random event) as a non-probabilistic basis for ranking protein families.
(DOCX)

## References

[1] Goater TM, Goater CP, Esch GW. Parasitisms: the Diversity and Ecology of Animal Parasites. Cambridge, UK: Cambridge University Press; 2014.

[2] Kabayo JP. Aiming to eliminate tsetse from Africa. Trends Parasitol. 2002;18: 473–475. 10.1016/S1471-4922(02)02371-1 12473355

[3] WHO Human African Trypanosomiasis. [cited 5 Nov 2019]. Available: http://www.who.int/trypanosomiasis_african/en/

[4] Franco JR, Simarro PP, Diarra A, Jannin JG. Epidemiology of human African trypanosomiasis. Clin Epidemiol. 2014;6: 257–275. 10.2147/CLEP.S39728 25125985 PMC4130665

[5] World Health Organisation. Investing to overcome the global impact of neglected tropical diseases: third WHO report on neglected diseases. 2015. ISBN 978 92 4 156486 1

[6] Clarkson MJ. Trypanosomes. Vet Parasitol. 1976;2: 9–29.

[7] Kappagoda S, Singh U, Blackburn BG. Antiparasitic Therapy. Mayo Clin Proc. 2011;86: 561–583. 10.4065/mcp.2011.0203 21628620 PMC3104918

[8] Baker N, de Koning HP, Mäser P, Horn D. Drug resistance in African trypanosomiasis: the melarsoprol and pentamidine story. Trends Parasitol. 2013;29: 110–118. 10.1016/j.pt.2012.12.005 23375541 PMC3831158

[9] World Health Organisation. Research priorities for Chagas disease, human African trypanosomiasis and leishmaniasis. World Health Organ Tech Rep Ser. 2012. 978 92 4 120975 5 23484340

[10] Hansen BD. Trypanosoma gambiense: Membrane transport of amino acids. Exp Parasitol. 1979;48: 296–304. 10.1016/0014-4894(79)90112-7 477819

[11] Clark Southworth G, Read CP. Absorption of some amino acids by the haemoflagellate, Trypanosoma gambiense. Comp Biochem Physiol Part A Physiol. 1972;41: 905–911. 10.1016/0300-9629(72)90354-4 4402093

[12] Voorheis HP. Amino-acid transport in Trypanosoma brucei. Trans R Soc Trop Med Hyg. 1971;65: 241–242. 10.1016/0035-9203(71)90248-3

[13] Marchese L, Nascimento J, Damasceno F, Bringaud F, Michels P, Silber A. The Uptake and Metabolism of Amino Acids, and Their Unique Role in the Biology of Pathogenic Trypanosomatids. Pathogens. 2018;7: 36 10.3390/pathogens7020036 29614775 PMC6027508

[14] McCann PP, Pegg AE. Ornithine decarboxylase as an enzyme target for therapy. Pharmacol Ther. 1992;54: 195–215. 10.1016/0163-7258(92)90032-U 1438532

[15] Grishin N V., Osterman AL, Brooks HB, Phillips MA, Goldsmith EJ. X-ray Structure of Ornithine Decarboxylase from Trypanosoma brucei: The Native Structure and the Structure in Complex with α-Difluoromethylornithine. Biochemistry. 1999;38: 15174–15184. 10.1021/bi9915115 10563800

[16] Jacobs RT, Nare B, Phillips MA. State of the art in African trypanosome drug discovery. Curr Top Med Chem. 2011;11: 1255–74. 10.2174/156802611795429167 21401507 PMC3101707

[17] Stibbs HH, Seed JR. Chromatographic evidence for the synthesis of possible sleep-mediators in Trypanosoma brucei gambiense. Experientia. 1973;29: 1563–1565. 10.1007/BF01943919 4543926

[18] Stibbs HH, Seed JR. Short-Term Metabolism of [14C]Tryptophan in Rats Infected with Trypanosoma brucei gambiense. J Infect Dis. 1975;131: 459–462. 10.1093/infdis/131.4.459 1117200

[19] Seed JR, Hall JE, Sechelski J. Phenylalanine metabolism in Microtus montanus chronically infected with Trypanosoma brucei gambiense. Comp Biochem Physiol B. 1982;71: 209–15. 10.1016/0305-0491(82)90242-5 7037281

[20] Sawalhy A, Seed JR, Attar H, Hall JE. Catabolism of Tryptophan by Trypanosoma evansi. J Eukaryot Microbiol. 1995;42: 684–690. 10.1111/j.1550-7408.1995.tb01616.x 8520582

[21] Seed JR, Hall JE, Price CC. A physiological mechanism to explain pathogenesis in African trypanosomiasis. Contrib Microbiol Immunol. 1983;7: 83–94. Available: http://www.ncbi.nlm.nih.gov/pubmed/6337778 6337778

[22] Newport GR, Page CR, Ashman PU, Stibbs HH, Seed JR. Alteration of Free Serum Amino Acids in Voles Infected with Trypanosoma brucei gambiense. J Parasitol. 1977;63: 15 10.2307/3280098 321737

[23] El Sawalhy A, Seed JR, Hall JE, El Attar H. Increased excretion of aromatic amino acid catabolites in animals infected with Trypanosoma brucei evansi. J Parasitol. 1998;84: 469–73. Available: http://www.ncbi.nlm.nih.gov/pubmed/9645841 9645841

[24] Hall JE, Seed JR. Increased urinary excretion of aromatic amino acid catabolites by Microtus montanus chronically infected with Trypanosoma brucei gambiense. Comp Biochem Physiol Part B Comp Biochem. 1984;77: 755–60. 10.1016/0305-0491(84)90309-2 6375946

[25] Hall JE, Seed JR, Sechelski JB. Multiple alpha-keto aciduria in Microtus montanus chronically infected with Trypanosoma brucei gambiense. Comp Biochem Physiol B. 1985;82: 73–8. Available: http://www.ncbi.nlm.nih.gov/pubmed/3902349 3902349 10.1016/0305-0491(85)90130-0

[26] McGettrick AF, Corcoran SE, Barry PJG, McFarland J, Crès C, Curtis AM, et al Trypanosoma brucei metabolite indolepyruvate decreases HIF-1α and glycolysis in macrophages as a mechanism of innate immune evasion. Proc Natl Acad Sci. 2016;113: E7778–E7787. 10.1073/pnas.1608221113 27856732 PMC5137691

[27] Hirumi H, Hirumi K. Continuous cultivation of Trypanosoma brucei blood stream forms in a medium containing a low concentration of serum protein without feeder cell layers. J Parasitol. 1989;75: 985–9. 10.2307/3282883 2614608

[28] Brun R, Schönenberger. Cultivation and in vitro cloning or procyclic culture forms of Trypanosoma brucei in a semi-defined medium. Short communication. Acta Trop. 1979;36: 289–92. Available: http://www.ncbi.nlm.nih.gov/pubmed/43092 43092

[29] Hunter KJ, Quesne SA Le, Fairlamb AH. Identification and Biosynthesis of N1,N9-Bis(Glutathionyl)Aminopropylcadaverine (Homotrypanothione) in Trypanosoma Cruzi. Eur J Biochem. 1994;226: 1019–1027. 10.1111/j.1432-1033.1994.t01-1-01019.x 7813456

[30] Kamleh A, Barrett MP, Wildridge D, Burchmore RJS, Scheltema RA, Watson DG. Metabolomic profiling using Orbitrap Fourier transform mass spectrometry with hydrophilic interaction chromatography: a method with wide applicability to analysis of biomolecules. Rapid Commun Mass Spectrom. 2008;22: 1912–1918. 10.1002/rcm.3564 18470888

[31] Creek DJ, Jankevics A, Burgess KE V., Breitling R, Barrett MP. IDEOM: an Excel interface for analysis of LC–MS-based metabolomics data. Bioinformatics. 2012;28: 1048–1049. 10.1093/bioinformatics/bts069 22308147

[32] Sumner LW, Amberg A, Barrett D, Beale MH, Beger R, Daykin CA, et al Proposed minimum reporting standards for chemical analysis. Metabolomics. 2007;3: 211–221. 10.1007/s11306-007-0082-2 24039616 PMC3772505

[33] Haug K, Cochrane K, Nainala VC, Williams M, Chang J, Jayaseelan KV, et al MetaboLights: a resource evolving in response to the needs of its scientific community. Nucleic Acids Res. 2019 10.1093/nar/gkz1019 31691833 PMC7145518

[34] Gloaguen Y, Morton F, Daly R, Gurden R, Rogers S, Wandy J, et al PiMP my metabolome: an integrated, web-based tool for LC-MS metabolomics data. Wren J, editor. Bioinformatics. 2017;33: 4007–4009. 10.1093/bioinformatics/btx499 28961954 PMC5860087

[35] Smith DRM, Willemse T, Gkotsi DS, Schepens W, Maes BUW, Ballet S, et al The First One-Pot Synthesis of L-7-Iodotryptophan from 7-Iodoindole and Serine, and an Improved Synthesis of Other L-7-Halotryptophans. Org Lett. 2014;16: 2622–2625. 10.1021/ol5007746 24805161

[36] Chakrabarti AC. Permeability of membranes to amino acids and modified amino acids: Mechanisms involved in translocation. Amino Acids. 1994;6: 213–229. 10.1007/BF00813743 11543596

[37] Mathieu C, Salgado AG, Wirdnam C, Meier S, Grotemeyer MS, Inbar E, et al Trypanosoma brucei eflornithine transporter AAT6 is a low-affinity low-selective transporter for neutral amino acids. Biochem J. 2014;463: 9–18. 10.1042/BJ20140719 24988048

[38] Santiveri CM, Jiménez MA. Tryptophan residues: Scarce in proteins but strong stabilizers of β-hairpin peptides. Biopolymers. 2010;94: 779–790. 10.1002/bip.21436 20564027

[39] Berger LC, Wilson J, Wood P, Berger BJ. Methionine Regeneration and Aspartate Aminotransferase in Parasitic Protozoa. J Bacteriol. 2001;183: 4421–4434. 10.1128/JB.183.15.4421–4434.2001 11443076 PMC95336

[40] Marciano D, Llorente C, Maugeri DA, de la Fuente C, Opperdoes F, Cazzulo JJ, et al Biochemical characterization of stage-specific isoforms of aspartate aminotransferases from Trypanosoma cruzi and Trypanosoma brucei. Mol Biochem Parasitol. 2008;161: 12–20. 10.1016/j.molbiopara.2008.05.005 18602174

[41] Azema L, Claustre S, Alric I, Blonski C, Willson M, Perié J, et al Interaction of substituted hexose analogues with the Trypanosoma brucei hexose transporter. Biochem Pharmacol. 2004;67: 459–467. 10.1016/j.bcp.2003.09.005 15037198

