# Supplementary material for: Halogenated tryptophan derivatives disrupt essential transamination mechanisms in bloodstream form Trypanosoma brucei
Source: PLoS Negl Trop Dis. 2020 Dec 4;14(12):e0008928. doi: 10.1371/journal.pntd.0008928 (PMC7744056; doi:10.1371/journal.pntd.0008928)
Supplement: S1 Table — Mean half maximal growth inhibition (EC50) values ±SD (μM) determined from n = 4 replicates. (DOCX) [file pntd.0008928.s001.docx]

S1 Table Bioactivity of tryptophan derivatives **1-7** and their methyl esters **8-14**, as well as natural tryptophan (**15**) and its methyl ester **16**, against T. brucei bloodstream form trypomastigotes, T. brucei procyclic form trypomastigotes, T. cruzi epimastigotes, L. Major promastigotes, and HeLa cells. Mean half maximal growth inhibition (EC_50_) values ±SD (μM) determined from n = 4 replicates.

| Free Acids | **1** | **2** | **3** | **4** | **5** | **6** | **7** | **15** |
| --- | --- | --- | --- | --- | --- | --- | --- | --- |
| *T. brucei BSF*^[[1]](#footnote-1)^a  (SI^e^) | >250  (nd) | >250  (nd) | 47.4  (>5.3) | >250  (nd) | >250  (nd) | 46.1  (>5.4) | 42.0  (>5.9) | >250  (nd) |
| *T. brucei* PCF^[[2]](#footnote-2)^b  (SI^e^) | >250  (nd) | >250  (nd) | 39.7  (>6.3) | >250  (nd) | 154.2  (>1.6) | 33.8  (>7.4) | 47.6  (>5.3) | >250  (nd) |
| *T. cruzi^[[3]](#footnote-3)^c*  (SI^e^) | >250  (nd) | >250  (nd) | >250  (nd) | >250  (nd) | >250  (nd) | >250  (nd) | >250  (nd) | >250  (nd) |
| *L. major^[[4]](#footnote-4)^d*  (SI^[[5]](#footnote-5)^e) | >250  (nd) | >250  (nd) | >250  (nd) | >250  (nd) | >250  (nd) | >250  (nd) | >250  (nd) | >250  (nd) |
| HeLa | >250 | >250 | >250 | >250 | >250 | >250 | >250 | >250 |
| Methyl Esters | **8** | **9** | **10** | **11** | **12** | **13** | **14** | **16** |
| *T. bruce BSFi*^a^  (SI^e^) | 149.5  (>1.7) | 157.9  (>1.6) | 3.5  (>71) | 101.7  (>2.5) | 139.7  (>1.8) | 3.7 (>67) | 2.7  (>92) | >250 (nd) |
| *T. brucei* PCF^[[6]](#footnote-6)^b  (SI^e^) | 82.8  (>3.0) | 81.2  (>3.1) | 3.1  (>81) | 77.2  (3.2) | 72.4  (3.5) | 1.8  (>139) | 1.5  (>167) | >250  (nd) |
| *T. cruzi*^b^  (SI^e^) | >250  (nd) | >250  (nd) | >250  (nd) | >250  (nd) | >250  (nd) | >250  (nd) | >250  (nd) | >250  (nd) |
| *L. major*^c^  (SI^e^) | >250  (nd) | >250  (nd) | >250  (nd) | >250  (nd) | >250  (nd) | >250  (nd) | >250  (nd) | >250  (nd) |
| HeLa | >250 | >250 | >250 | >250 | >250 | >250 | >250 | >250 |

1. a *Trypanosoma brucei brucei* bloodstream form trypomastigotes. [↑](#footnote-ref-1)
2. b *Trypanosoma brucei brucei* procyclic form trypomastigotes. [↑](#footnote-ref-2)
3. c *Trypanosoma cruzi* epimastigote form [↑](#footnote-ref-3)
4. d *Leishmania major* promastigote form [↑](#footnote-ref-4)
5. e Selectivity Index ([EC_50_ mammalian cells] / [EC_50_ parasite cells]) [↑](#footnote-ref-5)
6. [↑](#footnote-ref-6)
