# Supplementary material for: Halogenated tryptophan derivatives disrupt essential transamination mechanisms in bloodstream form Trypanosoma brucei
Source: PLoS Negl Trop Dis. 2020 Dec 4;14(12):e0008928. doi: 10.1371/journal.pntd.0008928 (PMC7744056; doi:10.1371/journal.pntd.0008928)
Supplement: S2 Table — Protein scores are derived from ions scores (−10log[P], where P is the probability that the observed match is a random event) as a non-probabilistic basis for ranking protein families. (DOCX) [file pntd.0008928.s002.docx]

S2 Table. Proteomic analyses of control T. brucei BSF parasites (no drug). Proteins are ranked by MASCOT assigned protein score, with the top ten results being shown in black, and proteins with Cl-Trp modified peptides detected shown in red. Protein scores are derived from ions scores (−10log[P], where P is the probability that the observed match is a random event) as a non-probabilistic basis for ranking protein families.

| Rank | Accession no. | Description | Mass | Num. Sig. Matches | Num. Sig. Sequences | Score |
| --- | --- | --- | --- | --- | --- | --- |
| 1 | tr\|Q4GYY6\|Q4GYY6_TRYB2 | Tubulin beta chain | 50413 | 130 | 16 | 5303 |
| 2 | tr\|Q4GYY5\|Q4GYY5_TRYB2 | Tubulin alpha chain | 50383 | 86 | 16 | 4191 |
| 3 | tr\|Q38B42\|Q38B42_TRYB2 | Fructose-bisphosphate aldolase | 41558 | 75 | 19 | 3317 |
| 4 | tr\|Q38AV5\|Q38AV5_TRYB2 | Chaperonin Hsp60, mitochondrial | 59751 | 54 | 22 | 2258 |
| 5 | tr\|Q38BV6\|Q38BV6_TRYB2 | Enolase | 47133 | 49 | 18 | 2120 |
| 6 | tr\|Q389P1\|Q389P1_TRYB2 | Heat shock protein 83 | 81169 | 56 | 20 | 2108 |
| 7 | tr\|Q383E5\|Q383E5_TRYB2 | Heat shock protein 70 | 75719 | 57 | 24 | 2018 |
| 8 | tr\|Q38DE9\|Q38DE9_TRYB2 | Glycerol kinase, glycosomal | 57131 | 48 | 17 | 2002 |
| 9 | tr\|Q38BE4\|Q38BE4_TRYB2 | Elongation factor 2 | 95300 | 58 | 25 | 1920 |
| 10 | tr\|D6XDN4\|D6XDN4_TRYB2 | ATP-dependent 6-phosphofructokinase | 53997 | 56 | 16 | 1823 |
| … |  |  |  |  |  |  |
| 507 | tr\|Q38C46\|Q38C46_TRYB2 | Uncharacterized protein | 158114 | 1 | 1 | 20 |
| … |  |  |  |  |  |  |
