# Supplementary material for: Halogenated tryptophan derivatives disrupt essential transamination mechanisms in bloodstream form Trypanosoma brucei
Source: PLoS Negl Trop Dis. 2020 Dec 4;14(12):e0008928. doi: 10.1371/journal.pntd.0008928 (PMC7744056; doi:10.1371/journal.pntd.0008928)
Supplement: S3 Table — Proteins are ranked by MASCOT assigned protein score, with the top ten results being shown in black, and proteins with Cl-Trp modified peptides detected shown in red. Protein scores are derived from ions scores (−10log[P], where P is the probability that the observed match is a random event) as a non-probabilistic basis for ranking protein families. (DOCX) [file pntd.0008928.s003.docx]

S3 Table. Proteomic analyses of T. brucei BSF parasites treated with tryptophan methyl ester analogue **10**. Proteins are ranked by MASCOT assigned protein score, with the top ten results being shown in black, and proteins with Cl-Trp modified peptides detected shown in red. Protein scores are derived from ions scores (−10log[P], where P is the probability that the observed match is a random event) as a non-probabilistic basis for ranking protein families.

| Rank | | Accession no. | Description | Mass | Num. Sig.  Matches | Num. Sig. Sequences | Score |
| --- | --- | --- | --- | --- | --- | --- | --- |
| 1 | tr\|Q4GYY6\|Q4GYY6_TRYB2 | | Tubulin beta chain | 50413 | 100 | 10 | 3557 |
| 2 | tr\|Q38B42\|Q38B42_TRYB2 | | Fructose-bisphosphate aldolase | 41558 | 44 | 11 | 1964 |
| 3 | tr\|Q383E5\|Q383E5_TRYB2 | | Heat shock protein 70 | 75719 | 40 | 15 | 1576 |
| 4 | tr\|Q4GYY5\|Q4GYY5_TRYB2 | | Tubulin alpha chain | 50383 | 36 | 9 | 1573 |
| 5 | tr\|Q38C42\|Q38C42_TRYB2 | | Phosphotransferase | 51776 | 38 | 10 | 1565 |
| 6 | tr\|Q38C41\|Q38C41_TRYB2 | | Phosphotransferase | 51630 | 36 | 10 | 1431 |
| 7 | tr\|Q57UH2\|Q57UH2_TRYB2 | | 69 kDa paraflagellar rod protein | 69953 | 29 | 11 | 1299 |
| 8 | tr\|Q38BV6\|Q38BV6_TRYB2 | | Enolase | 47133 | 30 | 9 | 1274 |
| 9 | tr\|Q38DE9\|Q38DE9_TRYB2 | | Glycerol kinase, glycosomal | 57131 | 39 | 9 | 1266 |
| 10 | tr\|Q4FKN7\|Q4FKN7_TRYB2 | | Uncharacterized protein (Fragment) | 483570 | 34 | 8 | 1243 |
| … |  | |  |  |  |  |  |
| 483 | tr\|Q580B5\|Q580B5_TRYB2 | | Dynein heavy chain, putative | 513145 | 1 | 1 | 27 |
| … |  | |  |  |  |  |  |
| 491 | tr\|Q57V97\|Q57V97_TRYB2 | | Protein phosphatase 2C, putative | 47448 | 1 | 1 | 26 |
| … |  | |  |  |  |  |  |
| 666 | tr\|Q57W62\|Q57W62_TRYB2 | | Uncharacterized protein | 50297 | 1 | 1 | 15 |
| … |  | |  |  |  |  |  |
| 675 | tr\|Q580H5\|Q580H5_TRYB2 | | Uncharacterized protein | 272385 | 1 | 1 | 14 |
| 676 | tr\|Q582T4\|Q582T4_TRYB2 | | Uncharacterized protein | 69143 | 1 | 1 | 14 |
| … |  | |  |  |  |  |  |
